# Association between *Mannheimia haemolytica* infection with reproductive physiology and performance in small ruminants: A review

**DOI:** 10.14202/vetworld.2019.978-983

**Published:** 2019-07-06

**Authors:** Faez Firdaus Abdullah Jesse, Nur Azhar Amira, Kamarulrizal Mat Isa, Arsalan Maqbool, Naveed Mohamad Ali, Eric Lim Teik Chung, Mohd Azmi Mohd Lila

**Affiliations:** 1 Institute of Tropical Agriculture and Food Security, Universiti Putra Malaysia, 43400 Serdang, Selangor, Malaysia; 2 Department of Veterinary Clinical Studies, Faculty of Veterinary Medicine, Universiti Putra Malaysia, 43400 Serdang, Selangor, Malaysia; 3 Department of Animal Science, Faculty of Agriculture, Universiti Putra Malaysia, 43400 Serdang, Selangor, Malaysia

**Keywords:** *Mannheimia haemolytica*, performance, physiology, pneumonic pasteurellosis, reproductive, small ruminants

## Abstract

Mannheimiosis or pneumonic pasteurellosis commonly occurs in small ruminants. Mannheimiosis is caused by *Mannheimia haemolytica* (*M. haemolytica*) a Gram-negative coccobacillus producing acute febrile and infectious condition resulting in death of animal if not diagnosed and treated promptly. *M. haemolytica* serotype A2 is a commensal of the nasopharynx, gaining access to the lungs when host defenses are compromised by stress or infection in small ruminants. Till date, there is a vast literature and research that has been conducted on the pathogenesis of *M. haemolytica* invariably on respiratory system and its related immune system and mechanisms. From the clinical point of view, infection or diseases involving vital organs will systemically affect the production and performance of the infected animal. Therefore, there is a huge gap of knowledge and research to answer the question whether there is any association between *M. haemolytica* infection with reproductive physiology and performance in small ruminants and how it affects the productivity level. This review will explore the possibilities of involvement and new potential research to be carried out to determine the involvement of male and female reproductive system with *M. haemolytica* infection among small ruminants.

## Introduction

Mannheimiosis or pneumonic pasteurellosis continues to be the principal constraint to the progression of small ruminant production in the tropics. Pneumonia is a respiratory problem which commonly occurs in small ruminants [1,2]. Ovine and caprine are equally vulnerable when they are subjected to severe stress or harsh environmental conditions [1]. The main causative agent of mannheimiosis is the *Mannheimia haemolytica* (*M. haemolytica*) serotype A2 [3,4]. Climatic stress, poor nutrition, poor husbandry, and mainly transportation stress are the predisposing factors leading to immunosuppression that contributes to the development of pneumonic pasteurellosis in sheep and goats, leading to huge economic losses [2]. The incidence rate of pneumonia in ruminants ranges from 10% to 40%, whereas the mortality exceeds 20%, with higher percentages in young ruminants [5]. Although many factors can be involved, climatic factor is among the primary and crucial limiting factors in the development of animal production in warm regions [6]. It was reported that there is an increasing number of pneumonia cases, especially during rainy seasons [7]. The disease in its typical clinical form is highly infectious, acute febrile progression with severe fibrinopurulent bronchopneumonia, and septicemia [8,9]. Disease ridden animals may die in a few days of clinical manifestations, but survivors from the bacterium may become persistently infected [10,11].

*M. haemolytica* is a hemolytic, Gram-negative, non-motile, and coccobacillus-shaped bacterium [12-14]. Accurate diagnosis of *M. haemolytica* primarily relies on bacteriological examination, biochemical characteristics, biotyping, and serotyping of the isolates [15]. It is now evident that *M. haemolytica* which was formerly known as *Pasteurella haemolytica* (*P. haemolytica*) is the main causative agent of the disease, although a number of investigators still believes that *Pasteurella multocida* and *M. haemolytica* are both commensal dwellers of the upper respiratory tract and that their pathogenic entities are associated with severe outbreaks in ruminants [16-18]. Till date, studies have only shown that *M. haemolytica* is a commensal of the upper respiratory tract, but none of them were looking at the presence of *M. haemolytica* in the reproductive tract, thus suggesting a new area of knowledge to be explored.

The role of virulence factors in the pathogenicity of *M. haemolytica* has been reported in Marru *et al*. [19], where virulence factors are unswervingly involved in the alteration of the organism from commensal into a pathogenic entity that is usually responsible for promoting adhesion, colonization, and proliferation of the organism in animal tissues [20]. There are at least six components of *M. haemolytica* that have been identified as virulence factors. This includes the capsule, outer membrane proteins, adhesins, neuraminidase, endotoxin lipopolysaccharide (LPS), and exotoxic leukotoxin A (LktA). From all these, the Lkt is pivotal in the induction of pneumonia. The role of Lkt-mediated infiltration and destruction of neutrophils and other leukocytes may lead to the impairment of bacterial clearance and contributes to the development of fibrinous pneumonia. LPS may act synergistically with Lkt in enhancing its effects and contributing to the endotoxic activity [21].

The pathogenesis of small ruminant pneumonia in contributing to the reproductive failure in female animals is still in a gray area and the pathogenesis could still not be drafted as no detailed studies were designed to look into this area. From the knowledge known, when there is a uterine infection due to bacteria, it deranges the role of the uterus and similarly disrupts the potentiality of the ovary and the regulatory structures in the hypothalamus and pituitary gland. A recent study by Beena *et al*. [22] observed that numerous uterine infections in small ruminants initiated in the endometrium gave negative effects toward breeding, pregnancy, or postpartum uterine inactivity of the affected animals. A study by Moeller [23] stated that 30% of uterine infections contributing to abortion in goats were caused by bacteria infection origin and in accord with Shallali *et al*. [24], they stated that the female genital tracts of small ruminants and their reproductive performances were commonly affected by the infection of pathogenic bacteria. The difference observed in the pathological changes in reproductive system of small ruminants in numerous literature and studies may be due to the diversity of the causative agent and relates to different severity of infection [25].

Studies involving mannheimiosis in small ruminants have reported valuable information on the clinical signs, gross lesions, and histopathological changes focusing on the respiratory system. However, knowledge of the pathophysiology changes due to this disease in the reproductive system has yet to be uncovered. Therefore, this review will explore the possible effects of *M. haemolytica* and its LPS on the reproductive physiology of small ruminants during or at post-infection. As stated by Timsit *et al*. [26], more researches are needed to reveal a holistic understanding of the pathogenesis and immunology of the bacterial infection as the information on these will play a significant role in getting a better understanding and knowledge of the pathogenesis and severity of *M. haemolytica* and its LPS infection.

## Effect of Bacteria and its LPS Infection on Male Reproductive System and its Physiology

The cell wall of *M. haemolytica* contains LPS, an endotoxin that has been identified as one of the major virulence factors that involve in the pathogenesis of pneumonic mannheimiosis [27-30]. Experimental evidence indicated that *M. haemolytica* endotoxin is directly toxic to the endothelial cells and capable of altering leukocyte functions and causes lysis of blood platelets [31]. A study was conducted by Abdullah *et al*. [32] to observe the pathological alterations in pituitary glands of animals challenged with *P. multocida* type B:2 and its LPS and the results revealed significant cellular changes such as edema, hemorrhage, degeneration, and necrosis in pituitary glands of the challenged animals.

Endotoxin from Gram-negative bacteria may give a negative effect on the male reproductive system of the affected animal. Olson and Brown [33] have explained that endotoxins are highly toxic substances that will exert a negative effect on the genital and visceral organs of bucks and rams with severe degenerative changes in testicles. Wallgren *et al*. [34] reported sperm abnormalities in the form of proximal protoplasmic droplets, strong coiled tail, acrosomal abnormalities, knobbed acrosome, and loose acrosome in small ruminants may due to endotoxin effect. Furthermore, Wallgren *et al*. [35] then reported a significant increase in abnormal sperm morphology in boars challenged with endotoxin extracted from *Salmonella typhimurium* where the abnormalities observed due to direct or indirect effect of the endotoxin were rise in abnormal sperm heads that reflect the presence of degeneration in the seminiferous epithelium. Bonte *et al*. [36] also observed abnormalities of cytoplasmic droplets and coiled tails of sperm as the signs of epididymal dysfunction in rams due to *Escherichia coli* (*E. coli*) endotoxins effect.

Bacterial infections in small ruminants, particularly in bucks, affect the overall health of the affected animal and may contribute toward the decrease in reproductive viability where a study by Khuder [37] in bucks challenged with bacteria and its toxin leads to a significant decrease in testosterone hormone concentration, decreased in semen quality, and a reduction in scrotal circumferences of the affected bucks. This is supported by Menkveld and Kruger [38] as they stated that the presence of bacterial infection in the male genitourinary tract has been correlated with the decreased in the number of sperm and abnormal morphology of sperm. A study conducted by Danek [39] in stallion challenged with *E. coli* endotoxin observed significant changes in the sperm motility, increased in the abnormal morphology of sperm such as the presence of cytoplasmic droplet in distal position, a single or loose tail loop, dwarf, and gigantic sperm heads after 9 weeks of post-LPS challenged. Diemer *et al*. [40] in their study have revealed that *E. coli* infection exerts negative effect toward sperm motility where it significantly lowered their progressive movement *in vitro*. This may due to the pathogenicity of *E. coli* that are related to the adhesive properties which may lead to inactivation of and damage to the sperm acrosomal reaction. Furthermore, another study by Diemer *et al*. [41] showed changes in sperm structure involving cell membranes of the head and midpiece including the internal and external acrosomal membranes due to bacterial infection. A study by García-Alvarez *et al*. [42] then reported that there were significant semen alterations due to stress and pyrexia in the affected animals due to bacterial infection which may spread through blood circulation throughout the body system including the male reproductive system that includes the testes, epididymis, and accessory male sex glands including bulbourethral gland, prostate, and vesicular glands.

From literature reviewed above, they suggested that the endotoxin exerts negative effects on the male hormones, semen quality, and its associated male reproductive organs which may lead to failure of the male reproductive functions. There are still gray areas on the questions whether there will be negative effects on the male reproductive system and its physiology in small ruminants due to *M. haemolytica* and its LPS endotoxin infection. Therefore, further research is warranted in this area, in which the findings would contribute to the understanding one of the reproductive disorders in small ruminants that may be due to *M. haemolytica* and its LPS endotoxin infection.

## Effect of Bacterial and its LPS Infection on Female Reproductive System and its Physiology

In female animals, anterior pituitary gland secretes reproductive hormones such as follicle-stimulating hormone (FSH), luteinizing hormone (LH), and prolactin [43]. Pituitary sex hormones regulate several functions that are crucial for reproduction, including oogenesis and lactation [44]. Secretion of estradiol hormone is influenced by the positive feedback on the functions of the hypopituitary-ovarian axis for farm animals such that the secretion of LH is triggered by the adenohypophysis through the pre-ovulatory surge of estrogen hormone which is the most crucial process for ovulation and corpus luteum formation. Dhaliwal *et al*. [45] and Lewis [46] have stated that the low concentration of female reproductive hormones in goats will have stimulatory effects on phagocytic activity of neutrophils. Therefore, progesterone hormone is the main hormone that regulates and controls the estrous cycle in small ruminants.

Several studies have demonstrated that bacterial infection can lead to a negative effect on the female reproductive system and its physiology in ruminant animals. A study by Ibrahim *et al*. [47] showed significant detection and isolation of *P. multocida* B:2 from the ovary, oviduct, uterine horn, uterine body, vagina, and mammary glands of buffaloes infected with the bacteria of *P. multocida* B:2 through oral and subcutaneous infections. There were also significant pathological and cellular changes observed in the female reproductive system of infected buffaloes. Therefore, this study by Ibrahim *et al*. [47] revealed that there is involvement of female reproductive system of ruminants during the pathogenesis of bacterial infection. In addition, Abdullah *et al*. [2,32] showed that the LPS extracted from *P. multocida* type B:2 is able to show significant alterations in the pituitary glands, reproductive organs, and associated hormonal level of female mice inoculated orally [2]. The cellular changes observed were edema, hemorrhage, degeneration, and necrosis in the pituitary glands and ovaries of the experimental mice, whereas there were significant changes in the progesterone and estrogen concentrations.

Another study conducted by Marza *et al*. [48] on reproductive hormonal variations and adenohypophyseal lesions in pre-pubertal buffalo heifers infected with LPS extracted from *P. multocida* type B:2 demonstrated a significant decrease in the LH, FSH, progesterone, estradiol, and gonadotrophin-releasing hormones concentrations. In a different study by Othman *et al*. [49] using Gram-positive bacteria, *Corynebacterium pseudotuberculosis* (*C. pseudotuberculosis*) infections through intradermal, intranasal, and oral routes in non-pregnant female goats, significant cellular changes such as necrosis, congestion, inflammatory cell infiltration, and edema that varied in severity in ovaries, uterus, and iliac lymph nodes of the infected female goats were observed. This study has recognized that Gram-positive bacteria, *C. pseudotuberculosis* infection could predispose toward infertility in affected small ruminant animals.

Small ruminants of all ages are vulnerable to mannheimiosis as stated in García-Alvarez *et al*. [42] and there is a paucity of data on the potential role of mannheimiosis disease toward the reproductive ability of female goats and sheep [50]. To support, Shallali *et al*. [24] stated that the presence of potentially pathogenic bacteria in the female genital tract might adversely affect its reproductive performance and might influence the life span of corpora lutea of goats.

Till date, there is no study conducted to explore the effects of *M. haemolytica* on the female reproductive system and performance in female small ruminants during and post-infection of mannheimiosis. Therefore, further research is needed in this area to understand one of the reproductive disorders in small ruminants which may be due to *M. haemolytica* and its LPS endotoxin infection.

## Correlation of Pro-inflammatory Cytokines during Bacterial and its LPS Infection on Female Reproductive System and its Physiology

In bacterial diseases of small ruminants, both pro-inflammatory and anti-inflammatory cytokines could constitute to infections and cure where excessive generation of cytokines can contribute to tissue and organ damage while being crucial in the eradication of infections [51]. Therefore, an upsurge in cytokines such as interleukin (IL)-6, IL-8, IL-10, IL-18, interferon-y (IFN-y), and tumor necrosis factor α (TNF-α) might have some implications in diagnosis and therapy of infections.

TNF-α is one of the pro-inflammatory cytokines that are crucial in mediating the host immune and inflammatory responses. TNF-α is also affected by the release of endotoxin from Gram-negative bacterium and it has a wide array of functions including the activation of immune cells such as the macrophages and the maturation of dendritic cells. Numerous studies have demonstrated that treatment with endotoxin or LPS results in an increase of TNF-α production. Jursza-Piotrowska and Siemieniuch [52] had conducted a study to determine whether the epithelial or the stromal cells are responsible for the release of TNF-α. Their study has shown that the exposure of LPS on feline endometrial cell *in vitro* significantly increases the release of TNF-α by endometrial epithelial cells, but not the stromal cells. This suggests that TNF-α might have a protective role in response to infections from Gram-negative bacteria.

IFN-y is also another pro-inflammatory cytokine that is released following exposure to LPS. A study by Sohn *et al*. [53] on bovine polymorphonuclear neutrophil (PMN) leukocytes found that the LPS derived from *E. coli* increases the level of IFN-y *in vitro*. The secretion of IFN-y by the PMN depicts that it plays a protective role during infection by Gram-negative bacteria by releasing the cytokine. Kim *et al*. [54] in their study found that the pro-inflammatory cytokines, particularly IL-1β, IL-6, and TNF-α concentration in the uterine flush from dairy cows with clinical or subclinical endometritis infections were elevated. Fischer *et al*. [55] also stated that the pro-inflammatory factor transcripts in bovine endometrial epithelial cells are regulated during the estrous cycle and are elevated in cases of subclinical or clinical endometritis. Xu *et al*. [56] also described that five pro-inflammatory mediators including three chemokine cytokine-induced neutrophil chemoattractant and IL-1a were increased on the stimulation of bacteria during endometritis.

However, there is very limited literature on pro-inflammatory cytokine responses in male ruminant animals during bacterial infections. Thus, information on *M. haemolytica* and its LPS affecting the pro-inflammatory cytokines concentration closely related with male and female reproductive system and its physiology in small ruminants during or post mannheimiosis infection need to be explored.

## Gross Pathology and Cellular Changes due to *M. haemolytica* Infection in Small Ruminants

*M. haemolytica* resides in the nasopharynx and tonsils with the latter considered the major reservoir [21]. Pneumonic pasteurellosis is usually acknowledged as a severe febrile respiratory disease with fibrinopurulent bronchopneumonia and pleurisy [57]. The most obvious gross lesions can be observed in the infected small ruminants with pasteurellosis are “marbling” of the lung, presence of excess straw-colored fluid in the thoracic cavity, also presence of frothy exudates in the trachea, bronchi and cut surface of the lungs. Area of consolidation, distended intralobular septa, pleural adhesion, and encapsulated foci containing whitish material known as sequestra can also be observed [32,58]. Histopathological lesions such as hemorrhage, edema, necrosis, and white blood cell infiltration were observed in the lungs of diseased goats [59]. Odugbo *et al*. [60] observed that the areas of the lung, alveolar spaces, and bronchioles were filled with edema, fibrin, red blood cells, and dense collections of primary macrophages and neutrophils, and the alveoli were flooded with fibrin-rich exudates. Dassanayake *et al*. [61] stated that vasculitis with fibrin thrombi is frequently observed in the cases of moderate to severe pneumonia in small ruminants. Till date, no studies have described on the gross postmortem changes and cellular changes involving reproductive system and its associated lymph nodes due to mannheimiosis infection in small ruminants. Therefore, knowledge of this part involving the reproductive system needs to be explored and the information will lead to better understanding and knowledge of the pathogenesis and effects of *M. haemolytica* toward the reproductive physiology and function in small ruminants.

## Conclusion

Taken together, the previous studies mentioned above clearly suggests that Gram-negative bacteria and its LPS have the ability to exert negative effects on the infected animals by increasing the level of pro-inflammatory cytokines, altering the concentration of reproductive hormones, and inducing gross pathological changes which in return may affect their reproductive functions. However, further research is needed to determine whether *M. haemolytica* and its LPS play a role in affecting the reproductive performances of infected animals. Besides, developing new knowledge in this area, it might also help in understanding this economically important disease which would be useful in tailoring an effective strategy to improvise the herd health control protocol.

## Authors’ Contributions

FFAJ conceived and framed the main idea of this manuscript. The first draft was made by NAA and NMA, covering the sections for male while KMI and AM worked on the female sections of the manuscript. The first draft was read and corrected by FFAJ. ELTC, MAML, and FFAJ proofread the second draft and finalized the manuscript. All authors have read and approved the final manuscript.
